# Prognostic analysis of rectal cancer patients after neoadjuvant chemoradiotherapy: different prognostic factors in patients with different TRGs

**DOI:** 10.1007/s00384-024-04666-z

**Published:** 2024-06-19

**Authors:** Yuan-ling Tang, Dan-dan Li, Jia-yu Duan, Xin Wang

**Affiliations:** Division of Abdominal Tumor Multimodality Treatment, Department of Radiation Oncology, Cancer Center, State Key Laboratory of Biological Therapy, West China Hospital of Sichuan University, No. 37 Guoxue Lane, Wuhou District, Chengdu City, 610041 Sichuan Province China

**Keywords:** Rectal cancer, Neoadjuvant chemoradiotherapy, Tumor regression grade, Neoadjuvant rectal score

## Abstract

**Purpose:**

The extent of tumor regression varies widely among locally advanced rectal cancer (LARC) patients who receive neoadjuvant chemoradiotherapy (NCRT) followed by total mesorectal excision (TME). The purpose of this retrospectively study is to assess prognostic factors in LARC patients with NCRT, and further to analyze survival outcomes in patients with different tumor regression grades (TRGs).

**Methods:**

This study includes LARC patients who underwent NCRT and TME at our institution. We retrospectively analyzed the clinicopathological characteristics and survival of all patients, and performed subgroup analysis for patients with different TRGs. Survival differences were compared using the Kaplan-Meier method and the log rank test. Additionally, a multiple Cox proportional hazard model was used to identify independent prognostic factors.

**Results:**

The study included 393 patients, with 21.1%, 26.5%, 45.5%, and 6.9% achieving TRG 0, TRG 1, TRG 2, and TRG 3, respectively. The overall survival (OS) rate and disease-free survival (DFS) rate for all patients were 89.4% and 70.7%, respectively. Patients who achieved TRG 0–3 had different 5-year OS rates (96.9%, 91.1%, 85.2%, and 68.8%, *P* = 0.001) and 5-year DFS rates (80.8%, 72.4%, 67.0%, 55.8%, *P* = 0.031), respectively. Multivariate analyses showed that the neoadjuvant rectal (NAR) score was an independent prognostic indicator for both overall survival (OS) (HR = 4.040, 95% CI = 1.792–9.111, *P* = 0.001) and disease-free survival (DFS) (HR = 1.971, 95% CI = 1.478–2.628, *P* ˂ 0.001). In the subgroup analyses, the NAR score was found to be associated with DFS in patients with TRG 1 and TRG 2. After conducting multivariate analysis, it was found that ypT stage was a significant predictor of DFS for TRG 1 patients (HR = 4.384, 95% CI = 1.721–11.168, *P* = 0.002). On the other hand, ypN stage was identified as the dominant prognostic indicator of DFS for TRG 2 patients (HR = 2.795, 95% CI = 1.535–5.091, *P* = 0.001). However, none of these characteristics was found to be correlated with survival in patients with TRG 0 or TRG 3.

**Conclusion:**

NAR score, in particular, appears to be the most powerful prognostic factor. It is important to consider various prognostic predictors for patients with different TRGs.

**Supplementary Information:**

The online version contains supplementary material available at 10.1007/s00384-024-04666-z.

## Introduction

The standard therapy for locally advanced rectal cancer (LARC) is neoadjuvant chemoradiotherapy (NCRT) followed by total mesorectal excision (TME) [1, 2]. The response to NCRT varies among patients, which could affect further treatment decisions and prognosis of patients [3]. There are many ways to classify tumor response to NCRT, and pathological stage has always been considered as the simplest and most robust prognostic indicator of LARC [4]. Besides, carcinoembryonic antigen (CEA) [5], tumor differentiation [6], pathologic type, and perineural invasion status (PNI) [7–9] are also been confirmed to be main prognostic factors and were strongly correlated with patients outcomes. Recently, the neoadjuvant rectal (NAR) score has been recommended as a primary endpoint to assess preoperative treatment efficacy in clinical trials of rectal cancer [10]. The National Comprehensive Cancer Network (NCCN) guideline recommends tumor regression grade (TRG) should be a potential role to stratify tumor response to NCRT. TRG is defined as the ratio of fibro-inflammatory tissue to residual tumor cells, ranging from TRG 0 (pathologic complete response (pCR); no viable cancer cells) to TRG 3 (poor response; minimal or no regression, extensive residual cancer) [11]. It was reported that patients with TRG 0 have excellent local and distant disease control rates [12, 13]. However, less than one-third patients achieve TRG 0 after NCRT, and the majority of patients exhibit an intermediate response with varying residual tumor infiltration [14]. We wonder whether patients with different TRGs also have different prognosis.

Consequently, the purpose of this study is to assess the prognostic significance of clinicopathological features in patients with LARC treated with NCRT and TME. Furthermore, this study aims to compare the prognostic factors in patients with different TRGs.

## Methods

### Patients

Patients were included in the study if the following criteria were met: rectal adenocarcinoma proved with pathology, pretreatment clinical stage II or III (T3–T4, or N+) confirmed by MRI, received NCRT and TME in our hospital between 2010 and 2022. Patients with concurrent malignancy at another site were excluded. This study was approved by the institutional review board and adhered Strengthening the Reporting of Observational studies in Epidemiology (STROBE).

### Neoadjuvant chemoradiotherapy

Neoadjuvant chemoradiotherapy included standardized concurrent chemoradiotherapy and total neoadjuvant therapy (TNT). The neoadjuvant pelvic IMRT (intensity-modulated radiation therapy) or VMAT (volumetric modulated arc therapy) with dose of 50–50.4 Gy/25–28 fractionations were delivered. Standardized concurrent chemotherapy was administered using capecitabine. TNT included at least 4 cycles of CAPOX (capecitabine plus oxaliplatin) before TME. The intervals between the completion of radiotherapy and TME are 5–8 weeks.

### Clinicopathological factors

The following parameters were evaluated as potential prognostic factors in this study: age at the time of diagnosis (≥ 60 or < 60 years), gender, pathologic TNM stage, T downstaging, baseline CEA, NAR score, TRG, and the pathologic status of PNI, lymphovascular space invasion (LVI), and CRM. The NAR score was calculated using the formula [5 pN-3(cT-pT) + 12]^2^ / 9.61, and classified as low (˂8), middle (8–16), and high (>16) [15]. TRG of the surgical specimens was performed according to the guidelines of the NCCN. Additionally, T downstaging was defined as ypT stage lower than cT stage. The clinical T stage and N stage were evaluated by a radiologist with 5 years of experience on the pelvic MRI, and reviewed by a radiologist with 10 years of experience. In the event of any inconclusive result, a senior radiologist with 20 years of experience was asked to confirm it. All pathologic information was determined by a pathologist with 10 years of experience. If the results were uncertain, a second pathologist with 20 years of experience will be consulted to provide a decision. Any discrepancies will be resolved according to the consultation. In order to minimize the potential for information bias, all eligible patients were included within the specified time period. And in the absence of the primary effectiveness evaluation index due to the failure to observe the case data throughout the follow-up process, the worst observation carried forward (WOCF) can be employed to carry forward the data. Furthermore, the absence of secondary effectiveness evaluation indicators is not carried forward.

We first identified predictive factors for overall survival (OS) and disease-free survival (DFS) in all eligible patients, and then evaluated prognostic factors in patients with different TRGs.

### Statistical analysis

Data were summarized as frequencies and percentages for categorical variables and as means and ranges for continuous variables. Categorical variables were evaluated using the chi-square or Fisher’s exact test, as appropriate. Continuous variables were assessed via the analysis of variance (ANOVA) test. The correlation between variables was evaluated for statistical significance using regression test, as necessary. The primary endpoints of the analysis were DFS (time from the date of diagnosis to the date of local recurrence, distant metastasis, or death) and OS (time from the date of diagnosis to the date of death). The survival analysis was based on the comparison of the Kaplan-Meier model with the log rank test. Univariate and multivariate analyses were performed using the Cox proportional hazards regression model [backward elimination (conditional)]. Subgroup analyses were conducted according to TRGs. IBM SPSS Statistics Version26 was used for all analysis. The significance was settled at a *P*-value ˂ 0.05 as usual.

## Results

### Patient characteristics

A total of 393 patients underwent NCRT followed by TME in our hospital during this decade, of which 219 patients (55.7%) received TNT and anther 174 patients (44.3%) received standard concurrent chemoradiotherapy. The number and proportion of patients at all levels of TRG were as follows: TRG 0 (83, 21.1%), TRG 1 (104, 26.5%), TRG 2 (179, 45.5%), TRG 3 (27, 6.9%). The average NAR score was 11.60, ranging from 0.00 to 50.36, and for NAR score classification, 139 patients (35.4%) had low score, 166 (42.2%) had middle score, and 88 (22.4%) had high score. Most patients (291, 74.0%) achieved T downstaging after NCRT. The clinicopathological parameters of all patients are shown in Table 1.

**Table 1:** Clinicopathologic characteristics of all patient (*n*=393)

Clinicopathologic characteristics	No. of patients (%)
Age (*x* ± *s*) (year)	56.27±11.11
Age	
≤ 60	247 (62.8)
˃ 60	146 (37.2)
Sex	
Female	146 (37.2)
Male	247 (62.8)
Baseline CEA level	
˂ 5 mg/L	231 (58.8)
≥ 5 mg/L	156 (39.7)
Unknown	6 (1.5)
cT stage	
cT2	3 (0.8)
cT3	190 (48.3)
cT4	200 (50.9)
Preoperative AJCC stage	
II	73 (18.6)
III	320 (81.4)
pT stage	
pT0	83 (21.1)
pT1	21 (5.3)
pT2	90 (22.9)
pT3	181 (46.1)
pT4	18 (4.6)
pN status	
Positive	97 (24.7)
Negative	296 (75.3)
T downstaging	
Yes	291 (74.0)
No	102 (26.0)
TRG	
0	83 (21.1)
1	104 (26.5)
2	179 (45.5)
3	27 (6.9)
NAR score	
Low (˂8)	139 (35.4)
Middle (8–16)	166 (42.2)
High (˃16)	88 (22.4)
Pathologic PNI	
Positive	74 (18.8)
Negative	318 (80.9)
Unknown	1 (0.3)
Pathologic LVI	
Positive	31 (7.9)
Negative	361 (91.9)
Unknown	1 (0.3)
Pathologic CRM	
Positive (≤ 1mm)	21 (5.3)
Negative (˃ 1mm)	256 (65.1)
Unknown	116 (29.5)

Abbreviations: *CEA*, carcinoembryonic antigen; *TRG*, tumor regression grade; *NAR score*, neoadjuvant rectal score; *PNI*, perineural invasion; *LVI*, lymphovascular space invasion; *CRM*, circumferential resection margin

The characteristics of patients with different TRGs are summarized in Table 2. As TRGs increased, the pathologic stage and NAR score became worse, and the probability of PNI and LVI was also higher. The average NAR score for TRG 0, TRG 1, TRG 2, and TRG 3 patients was 0.94, 11.21, 15.56, and 19.61, respectively (*P* ˂ 0.001). Additionally, only five of the TRG 1 patients exhibited positive lymph nodes, while over half of the TRG 3 patients were found to have positive lymph nodes following surgery. About 20% and 30% of cases presented with pathologic positive lymph nodes in patients with TRG1 and TRG2, respectively. Moreover, among TRG 1 patients, 50% were dragonized with ypT1-2 and ypT3-4, respectively. In contrast, a significantly greater proportion of TRG 2 patients were diagnosed with ypT3-4 than ypT1-2 (70.9% vs. 29.1%). None of the TRG 0 patients showed PNI or LVI, but in patients with TRG 3, more than 40% of them developed PNI, and nearly 26% of them experienced LVI. Furthermore, almost all TRG 0 patients exhibited low NAR scores, whereas over half of TRG 3 patients demonstrated high NAR scores.

**Table 2:** Clinicopathologic characteristics of patients with different TRGs

Variables	No. of patients (%)				*P-*value
	TRG 0	TRG 1	TRG 2	TRG 3	
ALL	83	104	179	27	-
Age					
≤ 60	60 (72.3)	64 (61.5)	104 (58.1)	19 (70.4)	0.131
˃ 60	23 (27.7)	40 (38.5)	75 (41.9)	8 (29.6)	
Sex					
Female	35 (42.2)	35 (33.7)	66 (36.9)	10 (37.0)	0.695
Male	48 (57.8)	69 (66.3)	113 (63.1)	17 (63.0)	
Baseline CEA level					
≤ 5mg/L	53 (63.9)	67 (64.4)	96 (53.6)	15 (55.6)	0.210
˃ 5mg/L	27 (32.5)	37 (35.6)	80 (44.7)	12 (44.4)	
Unknown	3 (3.6)	0 (0.0)	3 (1.7)	0 (0.0)	
cT stage					
cT2	2 (2.4)	1 (1.0)	1 (0.6)	0 (0.0)	0.465
cT3	43 (51.8)	52 (50.0)	85 (47.5)	9 (33.3)	
cT4	38 (45.8)	51 (49.0)	93 (52.0)	18 (66.7)	
Preoperative AJCC stage					
II	20 (24.1)	7 (6.7)	25 (14.0)	5 (18.5)	0.168
III	63 (75.9)	97 (93.3)	154 (86.0)	22 (81.5)	
ypT stage					
pT0	83 (100.0)	0 (0.0)	0 (0.0)	0 (0.0)	˂0.001
pT1–pT2	0 (0.0)	57 (54.8)	52 (29.1)	2 (7.4)	
pT3–pT4	0 (0.0)	47 (45.2)	127 (70.9)	25 (92.6)	
ypN status					
pN−	78 (94.0)	83 (79.8)	124 (69.3)	11 (40.7)	˂0.001
pN+	5 (6.0)	21 (20.2)	55 (30.7)	16 (59.3)	
T downstaging					
Yes	83 (100.0)	80 (76.9)	110 (61.5)	18 (66.7)	˂0.001
No	0 (0.0)	24 (23.1)	69 (38.5)	9 (33.3)	
NAR score					
Low (˂ 8)	82 (98.8)	34 (32.7)	22 (12.3)	1 (3.7)	˂0.001
Middle (8–16)	1 (1.2)	53 (51.0)	101 (56.4)	11 (40.7)	
High (˃ 16)	0 (0.0)	17 (16.3)	56 (31.3)	15 (55.6)	
Pathologic PNI					
Positive	83 (100.0)	93 (89.4)	127 (70.9)	15 (55.6)	˂0.001
Negative	0 (0.0)	10 (9.6)	52 (29.1)	12 (44.4)	
Unknown	0 (0.0)	1 (1.0)	0 (0.0)	0 (0.0)	
Pathologic LVI					
Positive	82 (98.8)	101 (97.1)	158 (88.3)	20 (74.1)	˂0.001
Negative	1 (1.2)	2 (1.9)	21 (11.7)	7 (25.9)	
Unknown	0 (0.0)	1 (1.0)	0 (0.0)	0 (0.0)	
Pathologic CRM					
Positive (≤ 1 mm)	0 (0.0)	11 (10.6)	6 (3.4)	4 (14.8)	˂0.001
Negative (˃ 1mm)	80 (96.)	74 (71.2)	91 (50.8)	11 (44.4)	
Unknown	3 (3.6)	19 (18.3)	82 (45.8)	12 (40.7)	

Abbreviations: *CEA*, carcinoembryonic antigen; *TRG*, tumor regression grade; *NAR score*, neoadjuvant rectal score; *PNI*, perineural invasion; *LVI*, lymphovascular space invasion; *CRM*, circumferential resection margin

### Oncological outcomes of all patients

**Fig. 1:**
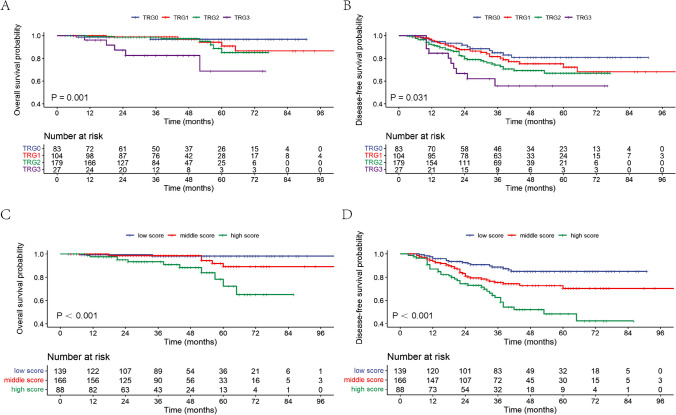
Survival curves of 393 patients with LARC after NCRT followed by TME surgery. **A** Correlation of TRG with 5-year OS rate. **B** Correlation of TRG with 5-year DFS rate. **C** Correlation of NAR score with 5-year OS rate. **D** Correlation of NAR score with 5-year DFS rate. TRG, tumor regression grade; NAR, neoadjuvant rectal; OS, overall survival; DFS, disease-free survival

Over a median follow-up period of 39 months (range, 3–128 months), the 5-year OS rate and 5-year DFS rate for all patients were 89.2% and 71.5%, respectively. Univariate analysis revealed that ypT stage, ypN stage, TRG, and NAR score were significant predictors for both OS and DFS, as shown in Table 3 and Fig. 1. Besides, positive CRM was associated with poor OS, and the presence of LVI and abnormal CEA had negative impacts on DFS. All significant variables from the univariate analysis were entered into a multivariate Cox regression model (Table 4). The results showed that the NAR score was an independent predictor of both DFS (HR = 1.971, 95% CI = 1.478–2.628, *P* ˂ 0.001) and OS (HR = 4.040, 95% CI = 1.792–9.111, *P* = 0.001). Patients with lower NAR scores showed superior long-term survival outcomes (low score vs. middle score vs. high score: 5-year OS rate: 98.2%, 88.8%, 72.1%; 5-year DFS rate: 87.1%, 70.1%, 48.4%).

**Table 3:** Univariate analyses for DFS and OS of all patients

Variables	5-year OS rate (%)	*P*-value	5-year DFS rate (%)	*P*-value
Age				
≤ 60	88.60	0.700	68.60	0.463
˃ 60	91.50		74.80	
Sex				
Female	94.10	0.091	76.60	0.101
Male	86.00		67.20	
Baseline CEA level				
≤ 5mg/L	89.10	0.603	76.30	0.030
˃ 5mg/L	94.30		63.70	
cT stage				
cT3	98.30	0.601	74.60	0.237
cT4	87.70		66.20	
Preoperative AJCC stage				
II	90.0	0.183	75.9	0.695
III	86.6		69.0	
ypT stage				
pT0	96.90	0.004	80.80	˂ 0.001
pT1–pT2	100.0		86.10	
pT3–pT4	78.70		57.20	
ypN status				
pN−	93.40	˂ 0.001	77.20	˂ 0.001
pN+	76.50		51.10	
T downstaging				
Yes	91.50	0.569	72.80	0.133
No	82.50		64.80	
TRG score				
0	96.90	0.001	80.80	0.031
1	91.10		72.40	
2	85.20		67.00	
3	68.80		55.80	
NAR score				
Low (˂8)	98.20	˂ 0.001	85.10	˂ 0.001
Middle (8–16)	89.40		70.50	
High (˃16)	72.30		48.40	
Pathologic PNI				
Positive	89.10	0.724	65.50	0.229
Negative	89.50		71.80	
Pathologic LVI				
Positive	70.10	0.146	50.30	0.006
Negative	90.30		72.50	
Pathologic CRM				
Positive (≤ 1 mm)	65.50	0.011	57.10	0.052
Negative (˃ 1 mm)	93.70		77.90	

Abbreviations: *CEA*, carcinoembryonic antigen; *TRG*, tumor regression grade; *NAR score*, neoadjuvant rectal score; *PNI*, perineural invasion; *LVI*, lymphovascular space invasion; *CRM*, circumferential resection margin

**Table 4:** Multivariate analyses for DFS and OS of all patients

Variables	OS		DFS	
	HR (95%CI)	*P*-value	HR (95%CI)	*P*-value
Baseline CEA level	-	-	1.432 (0.932–1.432)	0.096
ypT stage	1.077 (0.241–4.815)	0.576	1.541 (0.865–2.744)	0.293
ypN status	1.451 (0.149–14.106)	0.994	1.408 (0.599–3.311)	0.705
TRG score	1.581 (0.696–3.587)	0.329	0.845 (0.582–1.226)	0.890
NAR score	4.040 (1.792–9.111)	0.001	1.971 (1.478–2.628)	0.000
LVI	-	-	1.544 (0.820–2.906)	0.161
CRM	2.020 (0.442–9.239)	0.424	-	-

Abbreviations: *CEA*, carcinoembryonic antigen; *TRG*, tumor regression grade; *NAR score*, neoadjuvant rectal score; *LVI*, lymphovascular space invasion; *CRM*, circumferential resection margin; *HR*, hazard ratio; *CI*, confidential interval

### Risk factors for DFS and OS in patients with different TRGs

The 5-year OS rates for TRG 0–3 were 96.9%, 91.1%, 85.2%, and 68.8%, respectively (*P* = 0.001). The 5-year DFS rates also showed the same trend (80.8%, 72.4%, 67.0%, 55.8% for TRG 0–3, respectively, *P* = 0.031). There was no significant difference between patients with TRG 0 and TRG 1 in OS (*P* = 0.393) or DFS (*P* = 0.286). However, patients who showed highly sensitive to NCRT (TRG 0–1) had higher 5-year DFS rates than patients with low sensitivity to NCRT (TRG 2–3): 76.1% vs 65.7% (*P* = 0.021).

**Fig. 2:**
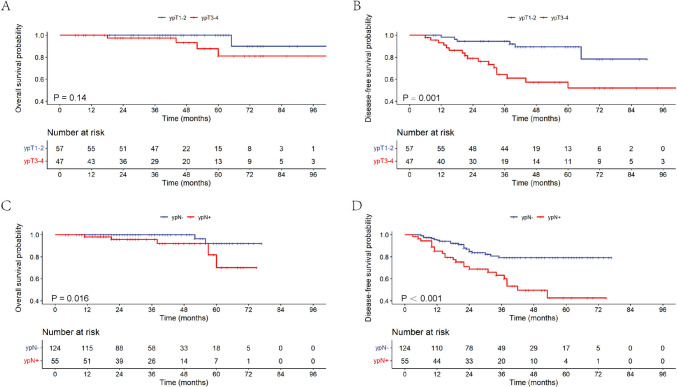
Survival curves of patients with different TRGs. **A** Correlation of ypT stage with 5-year OS rate in TRG 1 patients. **B** Correlation of ypT stage with 5-year DFS rate in TRG 1 patients. **C** Correlation of ypN stage with 5-year OS rate in TRG 2 patients. **D** Correlation of ypN stage with 5-year DFS rate in TRG 2 patients. TRG, tumor regression grade; OS, overall survival; DFS, disease-free survival

In the subgroup analyses of different TRGs, the results showed interesting (Fig. 2). In univariate analysis, NAR score, ypT stage, and ypN stage were all relevant prognostic predictors of DFS for patients with TRG 1 and TRG 2. While for TRG 0 and TRG 3 patients, none of these clinicopathologic showed correlation with clinical outcomes. In multivariate analysis, ypT stage was the independent factor of DFS for TRG1 patients (HR = 4.940, 95% CI = 1.802–13.544, *P* = 0.002), and ypN status manifested as the independent factor of DFS for TRG 2 patients (HR = 2.793, 95% CI = 1.533–5.086, *P* = 0.001).

## Discussion

Tumor response to NCRT can vary widely from complete absence of viable cancer cells in the primary site to total absence of tumor regression. In the current study, we investigated the prognostic impact of the clinicopathological features in LARC patients who underwent TME after NCRT, and the results showed that NAR score was the independent impact indicator for OS and DFS. However, subgroup analysis based on TRGs revealed different results. The independent factor for patients with TRG 1 was ypT stage, while for patients with TRG 2, ypN status was the dominant prognostic indicator. Moreover, for patients with TRG 0 and TRG 3, none of the clinical or pathologic characteristics was found to be associated with tumor outcomes. It is speculated that the reason for this may be due to the characteristics of patients with TRGs vary considerably. Patients with TRG 0 generally show favorable OS and DFS, while patients with TRG 3 are more likely to experience recurrence or metastasis. Another possible reason is that the number of patients with TRG 0 and TRG 3 is relatively small. In addition, we suspected that the pathological features of TRG 1 and TRG 2 patients may result in different prognostic factors. As most patients with TRG 1 did not present with positive lymph node, the prognostic impact of ypT stage was particularly significant. Conversely, most patients with TRG 2 were ypT3-4 stage, so the presence of positive lymph nodes had a crucial impact on outcomes. As far as we know, this is the first study to conduct a prognostic analysis of patients with varying TRGs.

Numerous published papers have mentioned the relation between TRG and patient’s outcome [16–18]. A cohort study showed significant differences in patient outcomes between any two TRG categories [19]. Previous reports indicate that 12–20% of patients achieved pCR after NCRT and had a favorable prognosis [20], which was similar to our study. However, excellent outcomes have also been found in patients with near pCR, and even comparable to those with pCR. Huh et al. [18] analyzed survival differences among various TRG grades, and they found that the 5-year OS rates of patients with TRG 0 and TRG 1 were similar (98% vs. 91%), and were significantly higher than that of patients with TRG 2 and TRG 3 (79%, *P* ˂ 0.001). Similar to their results, no significant difference was found in survival between patients with TRG 0 and TRG1 in our study (5-year OS rate: 96.9% vs. 90.9%, *P* = 0.381; 5-year DFS rate: 80.8% vs. 5.8%, *P* = 0.531), whereas high responders (patients with TRG 0–1) still had higher DFS than low responders (patients with TRG 2–3) (5-year DFS rate: 78.1% vs. 65.3%, *P* = 0.006). Likewise, Huebner et al. [21] also considered that partial pathologic response was a more superior predictor than pCR. However, inter-observer variation and different quantifying systems leaded to inconsistent results on the prognostic value of TRG [12]. Margherita and Hendrik et al. [22] suggested near pCR could not translate into good clinical prognosis.

Since the 1970s, the American Joint Committee on Cancer (AJCC) TNM staging system had been used worldwide for treatment selection in rectal cancer [23]. As some investigators have reported, pathologic TNM staging after surgery can provide valuable prognostic information on disease relapse and survival, and identify high-risk patients for additional postoperative adjuvant therapy [24–26]. Exactly, in our study, the ypT and ypN stage indeed played important role in the prognosis of TRG 1 and TRG 2 patients. Delitto et al. [27] indicated that the pathologic stage could dictate survival after NCRT for LARC patients. They found that stage III patients downstaged to ypT1N0 disease showed equivalent outcomes to patients with early-stage cT1N0 disease who underwent surgery directly. Cho et al. [28] identified the ypT stage and the presence of LVI as the independent prognostic indicators of pool DFS rates. Besides, the status of pathologic lymph nodes was considered to correlate with patient outcomes as well, in particular, those who developed with lateral lymph node (LNN) metastases generally had worse prognosis [29, 30]. In a study by Kim et al. [31], clinicopathologic factors were compared in 420 patients, and the results showed that ypN stage was the most important indicator for predicting DFS rather than TRG. In addition, Yokoyama et al. [32] demonstrated that the histology in pathologic positive lymph nodes may also be associated with survival, which could provide novel insights in LARC research.

One of the most noticeable changes before and after NCRT is T downstaging, which can be used to evaluate the tumor’s response to NCRT [33–35]. While some studies have suggested that T downstaging may not be associated with therapeutic efficacy or prognosis, it is important to consider the potential impact of this factor on patient outcomes [36, 37]. Mills et al. [38] found that T downstaging was not prognostically significant, while patients with TRG 3 were associated with inferior DFS; thus, they considered response categorization discrepancies may be partly explained by alternative patterns of residual disease, including tumor fragmentation. Similar to their conclusions, in the survival analysis of any group of patients, we did not find any correlation between T downstaging and OS, or DFS. Additionally, we found that ypT stage was the independent factor of DFS in patients with TRG 1, and it also demonstrated that the tumor response to neoadjuvant chemoradiotherapy may impact the prognosis of patients. It can be observed that, in the same situation of only a small proportion of residual tumor, patients with a concentric regression pattern (e.g., ypT1–2) have a more favorable prognosis than patients who with fragmentation pattern (e.g., ypT3–4).

The NAR score is another indicator reflecting the variation in tumor stage before and after NCRT, which is calculated based on the Valentini’s nomograms for OS [15]. The formula incorporated a weighted combination of the cT stage, ypT, and ypN stage. In the NSABP R-04 clinical trial, continuous NAR score has been validated to be significantly associated with OS. And NAR score was further classified as low (NAR < 8), middle (NAR = 8–16), and high (NAR > 16), and the results showed lower NAR score was associated with better OS. Moreover, NAR score has also been demonstrated to be a prognostic role and an ideal surrogate endpoint for DFS in the CAO/ARO/AIO-04 randomized phase 3 trial [10]. In addition, in a retrospective study of 1172 LARC patients, the NAR score was proven to outperform pCR in predicting OS. Meanwhile, many studies indicated that higher NAR scores tended to associated with fewer pCRs, lower TRGs, and more advanced pathologic stage, which from the side that [26, 39]. Our study found that higher NAR scores were associated with worse patient survival and were the most dominant factor. More interestingly, Sert et al. [40] not only evaluated the role of NAR scores in predicting outcomes, but also compared it with TRG. According to their research results, NAR score adds the value of tumor changes before and after NCRT in addition to ypT and ypN status. Therefore, it is suggested to use NAR score to predict patient survival.

There were some limitations of the present study that deserve consideration. Firstly, this was a single center retrospective study, and a prospective approach would be more valuable. Secondly, inter- and intra-observer variability of TRG classification was not taken into account, but this deficiency existed in most studies. Thirdly, the sample size is relatively small in subgroup analyses, which may result in a lack of statistical results.

In conclusion, this study evaluated the prognostic effects of different factors on long-term survival for LARC patients who received NCRT and TME. To date, there was no consensus on the effective predictors of prognosis for LARC patients received NCRT and TME. However, it is undeniable that the NAR score, TRG, and pathological stage are vital factors in prognostic evaluation, and patients with different TRGs may need to consider diverse prognostic predictors.

## Supplementary information

ESM 1 (PDF 125 kb)

## Data Availability

The data that support the findings of this study are available on request from the corresponding author, upon reasonable request. The data are not publicly available due to privacy or ethical restrictions.
