# Supplementary material for: Prognostic analysis of rectal cancer patients after neoadjuvant chemoradiotherapy: different prognostic factors in patients with different TRGs
Source: Int J Colorectal Dis. 2024 Jun 19;39(1):93. doi: 10.1007/s00384-024-04666-z (PMC11186864; doi:10.1007/s00384-024-04666-z)
Supplement: Supplementary file 1 — (PDF 125 kb) [file 384_2024_4666_MOESM1_ESM.pdf]

STROBE Statement—checklist of items that should be included in reports of observational studies

	Item No.	Recommendation	Page No.	Relevant text from manuscript
**Title and abstract**	1	(*a*) Indicate the study's design with a commonly used term in the title or the abstract	2	Line 3: The purpose of this retrospectively study xxx
		(*b*) Provide in the abstract an informative and balanced summary of what was done and what was found	2	Line 3-line 27
**Introduction**				
Background/rationale	2	Explain the scientific background and rationale for the investigation being reported	3	Line 3-line 26
Objectives	3	State specific objectives, including any prespecified hypotheses	3	Line 27-line 30: the purpose of this study is to assess the prognostic significance of clinicopathological features in patients with LARC treated with NCRT and TME. Furthermore, this study aims to compare the prognostic factors in patients with different TRGs.
**Methods**				
Study design	4	Present key elements of study design early in the paper	5	Line 8-line 10
Setting	5	Describe the setting, locations, and relevant dates, including periods of recruitment, exposure, follow-up, and data collection	4	xxx received NCRT and TME in our hospital between 2010 and 2022.
Participants	6	(*a*) *Cohort study*—Give the eligibility criteria, and the sources and methods of selection of participants. Describe methods of follow-up<br>*Case-control study*—Give the eligibility criteria, and the sources and methods of case ascertainment and control selection. Give the rationale for the choice of cases and controls<br>*Cross-sectional study*—Give the eligibility criteria, and the sources and methods of selection of participants	4	Line 3-line 6
		(*b*) *Cohort study*—For matched studies, give matching criteria and number of exposed and unexposed	-	Not Applicable

		Case-control study—For matched studies, give matching criteria and the number of controls per case		
Variables	7	Clearly define all outcomes, exposures, predictors, potential confounders, and effect modifiers. Give diagnostic criteria, if applicable	5	Line 8-line 10
Data sources/ measurement	8*	For each variable of interest, give sources of data and details of methods of assessment (measurement). Describe comparability of assessment methods if there is more than one group	4	Line 18-line 30
Bias	9	Describe any efforts to address potential sources of bias	5	Line 1-line 3
Study size	10	Explain how the study size was arrived at	4	Line 3-line 7, actually, this study was a retrospective study, and the patients were retrospectively collected from 2010 to 2022, in accordance with the inclusion criteria.

Continued on next page

Quantitative variables	11	Explain how quantitative variables were handled in the analyses. If applicable, describe which groupings were chosen and why	5	Line 12-line 15
Statistical methods	12	(*a*) Describe all statistical methods, including those used to control for confounding	5	Line 13-line 24
		(*b*) Describe any methods used to examine subgroups and interactions	5	Subgroups analyses were conducted according to TRGs.
		(*c*) Explain how missing data were addressed	5	Line 3-line 7
		(*d*) *Cohort study*—If applicable, explain how loss to follow-up was addressed<br>*Case-control study*—If applicable, explain how matching of cases and controls was addressed<br>*Cross-sectional study*—If applicable, describe analytical methods taking account of sampling strategy		
		(*e*) Describe any sensitivity analyses	-	Sensitive analyses were not involved in this study
**Results**				
Participants	13*	(a) Report numbers of individuals at each stage of study—eg numbers potentially eligible, examined for eligibility, confirmed eligible, included in the study, completing follow-up, and analysed	5	Line 27-line 29
		(b) Give reasons for non-participation at each stage	-	All eligible patients were enrolled in this study
		(c) Consider use of a flow diagram	-	Flow diagram were not involved in this study
Descriptive data	14*	(a) Give characteristics of study participants (eg demographic, clinical, social) and information on exposures and potential confounders	5-6	Line 27-line 30, and line 1-line 19, and Table 1
		(b) Indicate number of participants with missing data for each variable of interest	15	Table 1
		(c) *Cohort study*—Summarise follow-up time (eg, average and total amount)	6	Line 23: Over a median follow-up period of 39 months (range, 3-128 months) xxx
Outcome data	15*	*Cohort study*—Report numbers of outcome events or summary measures over time	-	Not Applicable
		Case-control study—Report numbers in each exposure category, or summary measures of exposure	-	Not Applicable
		Cross-sectional study—Report numbers of outcome events or summary measures	6	Line 23-line 24: the 5-year OS rate and 5-year DFS rate for all patients were 89.2% and 71.5%
Main results	16	(*a*) Give unadjusted estimates and, if applicable, confounder-adjusted estimates and their precision	6, 7	Line 23-line 30, and line 1-line 4

	(eg, 95% confidence interval). Make clear which confounders were adjusted for and why they were included		
	(*b*) Report category boundaries when continuous variables were categorized	15	Table 1
	(*c*) If relevant, consider translating estimates of relative risk into absolute risk for a meaningful time period	-	Not Applicable

Continued on next page

Other analyses	17	Report other analyses done—eg analyses of subgroups and interactions, and sensitivity analyses	7	Line 6-line 20
**Discussion**				
Key results	18	Summarise key results with reference to study objectives	7, 8	Line 22-line 30, and Line 1-line 11
Limitations	19	Discuss limitations of the study, taking into account sources of potential bias or imprecision. Discuss both direction and magnitude of any potential bias	10	Line 22-line 26
Interpretation	20	Give a cautious overall interpretation of results considering objectives, limitations, multiplicity of analyses, results from similar studies, and other relevant evidence	8, 9, 10	
Generalisability	21	Discuss the generalisability (external validity) of the study results	10	Line 27-line 30
**Other information**				
Funding	22	Give the source of funding and the role of the funders for the present study and, if applicable, for the original study on which the present article is based	18	Line 10-line 14

*Give information separately for cases and controls in case-control studies and, if applicable, for exposed and unexposed groups in cohort and cross-sectional studies.

Note: An Explanation and Elaboration article discusses each checklist item and gives methodological background and published examples of transparent reporting. The STROBE checklist is best used in conjunction with this article (freely available on the Web sites of PLoS Medicine at http://www.plosmedicine.org/, Annals of Internal Medicine at http://www.annals.org/, and Epidemiology at http://www.epidem.com/). Information on the STROBE Initiative is available at www.strobe-statement.org.
